# Clinical outcomes of 3T magnetic resonance imaging-guided lumbar and sacral foraminal injections

**DOI:** 10.1007/s00234-023-03234-6

**Published:** 2023-10-17

**Authors:** Pauline C. Guillemin, Rares Salomir, Nicolas Lauper, Orane Lorton, Enrique Maturana, Alex Stöckli, Pierre-Alexandre Poletti, Dennis E. Dominguez, Sana Boudabbous, Max Scheffler

**Affiliations:** 1 Faculty of Medicine, University of Geneva, Geneva, Switzerland; 2 Division of Radiology, Geneva University Hospitals, Geneva, Switzerland; 3 Division of Orthopedic Surgery and Traumatology, Geneva University Hospitals, Geneva, Switzerland; 4 Division of Radiology, Geneva University Hospitals, Thônex, Switzerland

**Keywords:** MRI-guidance, Radicular pain, Nerve root infiltration

## Abstract

**Purpose:**

This article evaluates the feasibility, safety, and efficacy of MRI-guided lumbar or sacral nerve root infiltration for chronic back pain. We compared the outcomes of our MRI-guided infiltrations with data from CT-guided infiltrations reported in the literature and explored the potential advantages of MRI guidance.

**Method:**

Forty-eight MRI-guided nerve root infiltrations were performed using a 3 T MRI machine. The optimal needle path was determined using breathhold T2-weighted sequences, and the needle was advanced under interleaved guidance based on breathhold PD-weighted images. Pain levels were assessed using a numeric rating scale (NRS) before the procedure and up to 5 months after, during follow-up. Procedure success was evaluated by comparing patients’ pain levels before and after the infiltration.

**Results:**

The MRI-guided infiltrations yielded pain reduction 1 week after the infiltration in 92% of cases, with an average NRS substantial change of 3.9 points. Pain reduction persisted after 5 months for 51% of procedures. No procedure-related complications occurred. The use of a 22G needle and reconstructed subtraction images from T2 FatSat sequences improved the workflow.

**Conclusion:**

Our study showed that MRI-guided nerve root infiltration is a feasible, safe, and effective treatment option for chronic back pain. Precise positioning of the needle tip and accurate distribution of the injected solution contributed to the effectiveness of MRI-guided infiltration, which appeared to be as accurate as CT-guided procedures. Further research is needed to explore the potential benefits of metal artifact reduction sequences to optimize chronic back pain management.

## Introduction

Low back pain is a multifactorial disease, and different treatment options are continuously discussed in the medical community [1]. The choice of the best therapeutic approach for a patient heavily relies on the accurate identification of etiologic factors and symptoms [2]. This applies in particular to the distinction between localized low back pain (lumbago) versus radicular pain. The latter typically follows the supply territory of a distinct nerve into the groin, the buttocks, or a lower limb. In the case of L4 to S3 nerve roots, the term sciatica is used for related symptoms of pain or paresthesia (numbness, burning, tingling sensation). Radicular pain is believed to be caused by nerve inflammation, sometimes induced by direct mechanical nerve compression [3], the most common cause being a bulging lumbar disc and/or spondylolisthesis [3–5]. Lower limb radiculopathy is a considerable worldwide health problem, without gender predominance. It has a lifetime incidence of 10 to 40% that affects patients’ quality of life, causes economic losses, and increases healthcare costs [3].

Lower back pain treatment follows a stepwise approach. The first step is to determine whether a motor deficit is present. Should this be the case, prompt surgical evaluation is indicated to precisely define its extent. In the absence of significant motor deficit, the second step usually involves patient education and adapted oral medication, including non-steroidal anti-inflammatory drugs and muscle relaxants. Other complementary second-line treatments include massages, physiotherapy, and acupuncture [6, 7]. Unlike lumbago care, the third step for radicular pain, if it lasts longer than 6–8 weeks, consists of lumbar or sacral nerve root infiltrations. These infiltrations provide pain relief and may also have a diagnostic purpose—the confirmation of a single affected nerve root—if surgery is required for the patient during the course of the disease. For inoperable patients, repeated infiltrations might even represent a final treatment option.

Several interventional approaches have been described for performing lumbar or sacral nerve root infiltrations. The needle route, the injected drugs, and image guidance techniques can be adapted on a case-specific basis.

The three main access routes in clinical practice are (1) direct epidural interlaminar access, (2) foraminal access, and (3) caudal access. If a foraminal access is chosen, the injected products may or may not diffuse medially into the intraspinal epidural space (so-called transforaminal epidural). A relatively lateral foraminal access may also be referred to as a “selective nerve block.” Effective pain relief has been shown for the foraminal approach in multiple studies [8–12]. The caudal approach is less frequently used for epidural injections [13]. In a study where patients had an underlying disc herniation, both foraminal and interlaminar access were equally effective [14]. In another study, the foraminal approach was more effective than the translaminar one at 6 months [15]. Some authors recommend foraminal access over translaminar access, because it allows to deploy the injected drugs as close as possible to the nerve root [16]. On the other hand, a translaminar access may be preferred over the transforaminal technique when several nerve roots are affected at the same time, for example, in a context of severe lumbar canal stenosis. An overall success rate of 76–88% has been reported in a review on foraminal nerve root infiltrations [12]. Brändle et al. studied interlaminar and foraminal infiltrations and found a pain reduction at 10 days in 72% of cases if the pain was related to lumbar disk herniation [17].

For most injections in the spinal region, a combination of an anti-inflammatory corticosteroid is advocated, in combination with a local anesthetic of the amino-amide type. Apart from intra-articular injections, the corticosteroid should preferably be non-particulate [16].

**Fig. 1:**
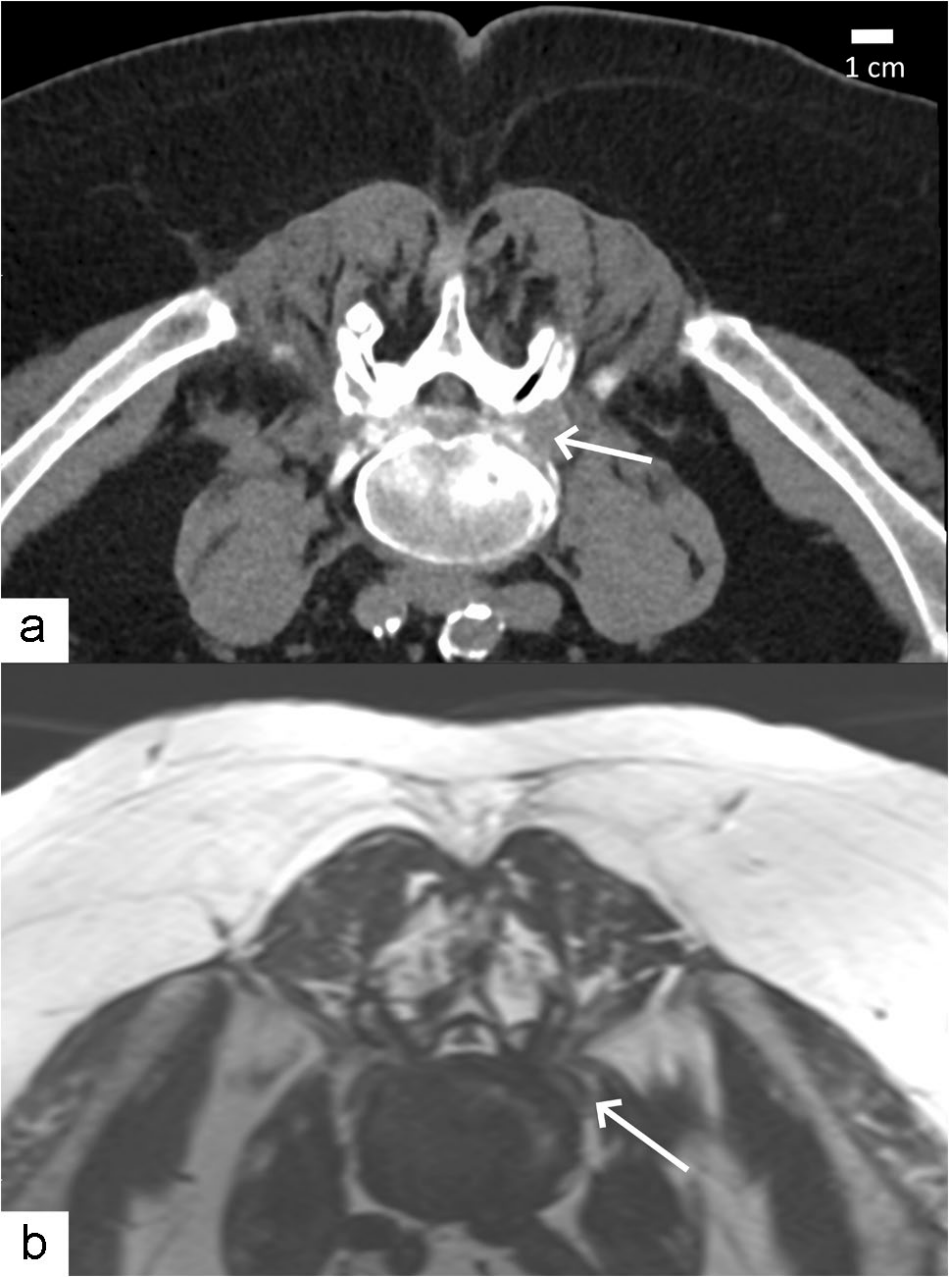
Non-contrast CT (**a**) and MRI (**b**) scans obtained in a 64-year-old man with left L5 radiculopathy, secondary to L5 antelisthesis, and severe L5-S1 neural foraminal narrowing. **a** Axial oblique CT image showing fibrotic changes inside the neural foramen (arrow), thwarting a clear delineation of exiting nerve. **b** Axial oblique proton density-weighted MRI image clearly depicting the nerve (arrow)

The foraminal infiltration technique has been largely described under CT [17–19] and fluoroscopic guidance [20]. Infiltrations are less frequently performed under MRI guidance, because of higher costs and longer machine time. For instance, the foraminal procedure takes an estimated 20–30 min under CT at our institution. However, MRI guidance may be advantageous in specific cases for several reasons. First, radioprotection can be an issue if the procedure is repeated, performed in a younger patient or child, or during pregnancy, when the uterus is normally within the imaged volume. Second, MRI-guided interventions do not require injection of iodine-based contrast agent—routinely used if the guidance modality is CT and systematically used under fluoroscopy—which is sometimes contraindicated or can cause allergic reactions. Air can be used as an alternative if iodinated contrast media are contraindicated, as shown in 1000 cases by Chang et al*.* [21]. However, iodinated contrast media remain part of the standard protocol in many institutions. Instead, MRI guidance can be assisted by intra-operatory delivery at the infiltration site of diluted gadolinium-containing contrast media, or simply normal saline serum, as in our own experience [22]. Furthermore, MRI allows for straightforward planning of an oblique access, for example, in cases of lumbo-sacral transitional anomalies. Not all CT scanners offer gantry tilting, or operators might not be familiar with the technique, which impedes visualization of direct access to the nerve root due to the strict axial plane. Lastly, MRI offers improved soft-tissue contrast which might be necessary in patients with fibrotic changes in the neural foramen [23–25], as illustrated in Fig. 1. In fact, if the amount of fat surrounding the nerve root is reduced, it can be more difficult to localize under CT. On the other hand, fluoroscopy is even more different because soft tissues are not depicted. After the description of the MRI-guided infiltration technique by other groups at 1 T and 1.5 T field strengths [24, 26], our group has reported the optimization steps at 3 T and demonstrated that the procedure was safe and precise [22].

The aim of the current report was to report further updates and improvements to the technical and clinical workflows on 48 procedures, to evaluate responses to infiltration up to 5 months of follow-up, and to perform a comparison with CT-guided intervention data available in the literature.

## Materials and methods

### Study design

Forty-eight MRI-guided nerve root infiltrations were carried out in 40 patients between November 2017 and July 2022, on a 3 T MRI machine (Magnetom Skyra, Siemens Healthcare, Erlangen, Germany). As described in a former publication [22], an adapted semi-flexible surface coil was used in addition to the standard spine coil.

### Ethical approval

Study approval was obtained from the ethics committee of the CCER from Geneva (n°2019–02301) and all methods were performed in accordance with the relevant guidelines and regulations. All patients were informed about the study before the intervention and gave their written informed consent to participate in the study.

### Patient selection

The inclusion criteria were patients with lumbar or sacral radiculopathy selected for MRI-guided lumbar or sacral nerve root infiltrations after multidisciplinary board discussion involving radiology, neurosurgery, rheumatology, orthopedic surgery, and pain relief teams. Patients were assessed clinically by a spinal surgeon and by standard musculoskeletal MRI using sagittal T1 and T2 turbo spin echo (TSE), axial T2 TSE, and sagittal T2 TSE FatSat Short-TI Inversion Recovery (STIR) sequences. Other tests such as electromyography were not routinely performed since the diagnostic value of the intervention was considered sufficiently high.

The exclusion criteria included all contraindications for MRI examinations, in particular claustrophobia and presence of metallic devices. These conditions could be ruled out already at the step of the standard before the intervention MRI mentioned above.

In this study, no pregnant women were recruited for infiltration. Therefore, we cannot provide specific advice regarding the use of the technique in this particular population.

### Intervention

**Fig. 2:**
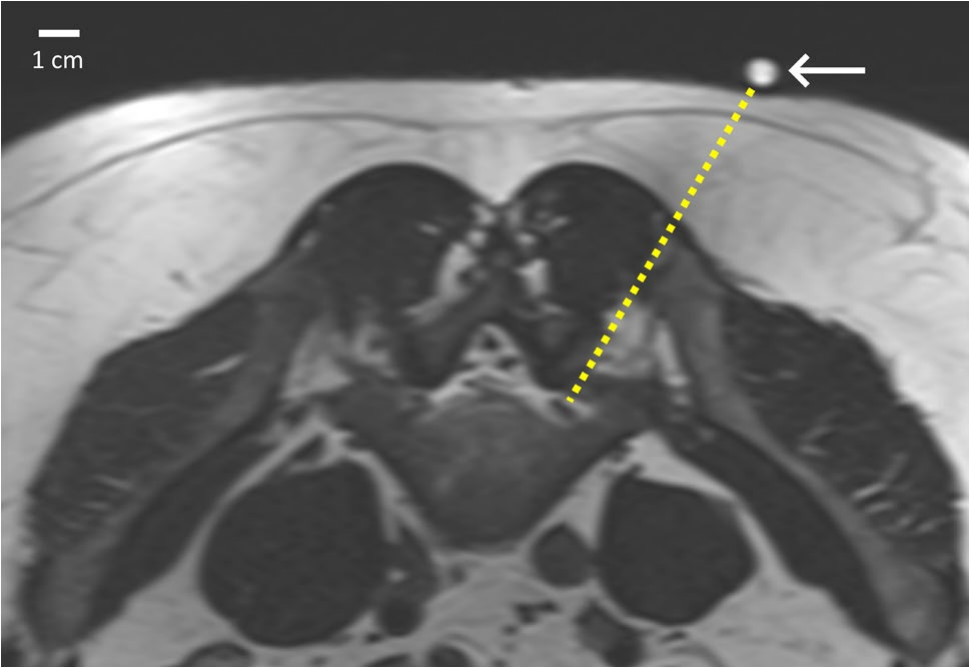
Axial T2-weighted MRI image of 43-year-old man with suspected left L5 radiculopathy in a context of congenital lumbar canal stenosis and L4–L5 discal hernia. A silicon marker (arrow) has been placed on the skin for planification of needle access (dotted line)

In the first step, the optimal needle path was determined by a radiologist using an axial breathhold T2-weighted TSE sequence, repeated a second time for confirmation after placement of a small silicone marker on the chosen skin puncture point (Fig. 2). Infiltration of a 1% lidocaine solution via 25G and 22G needles was performed for superficial and deep anesthesia.

A breathhold PD-weighted sequence was then used to repeatedly control the advancement of a 20G or 22G MRI-compatible needle with a stylet (Cytocut MRI, MDL, Delebio, Italy) until the needle came to lie directly behind the exiting nerve root in the neural foramen or a sacral foramen.

On May 20, 2022, a 22G needle built from identical alloy was employed as an alternative to the standard 20G needle to compare the size of artifacts. The assessment criterion focused on visual artifacts, specifically evaluating the width of the dark band that artificially increased the apparent width of the needle as described in Scheffler et al. [22]. It is important to note that the 22G needle diameter is smaller than that of the 20G needle; therefore, the spatial extension of the magnetic field perturbation is proportionally reduced [27].

**Fig. 3:**
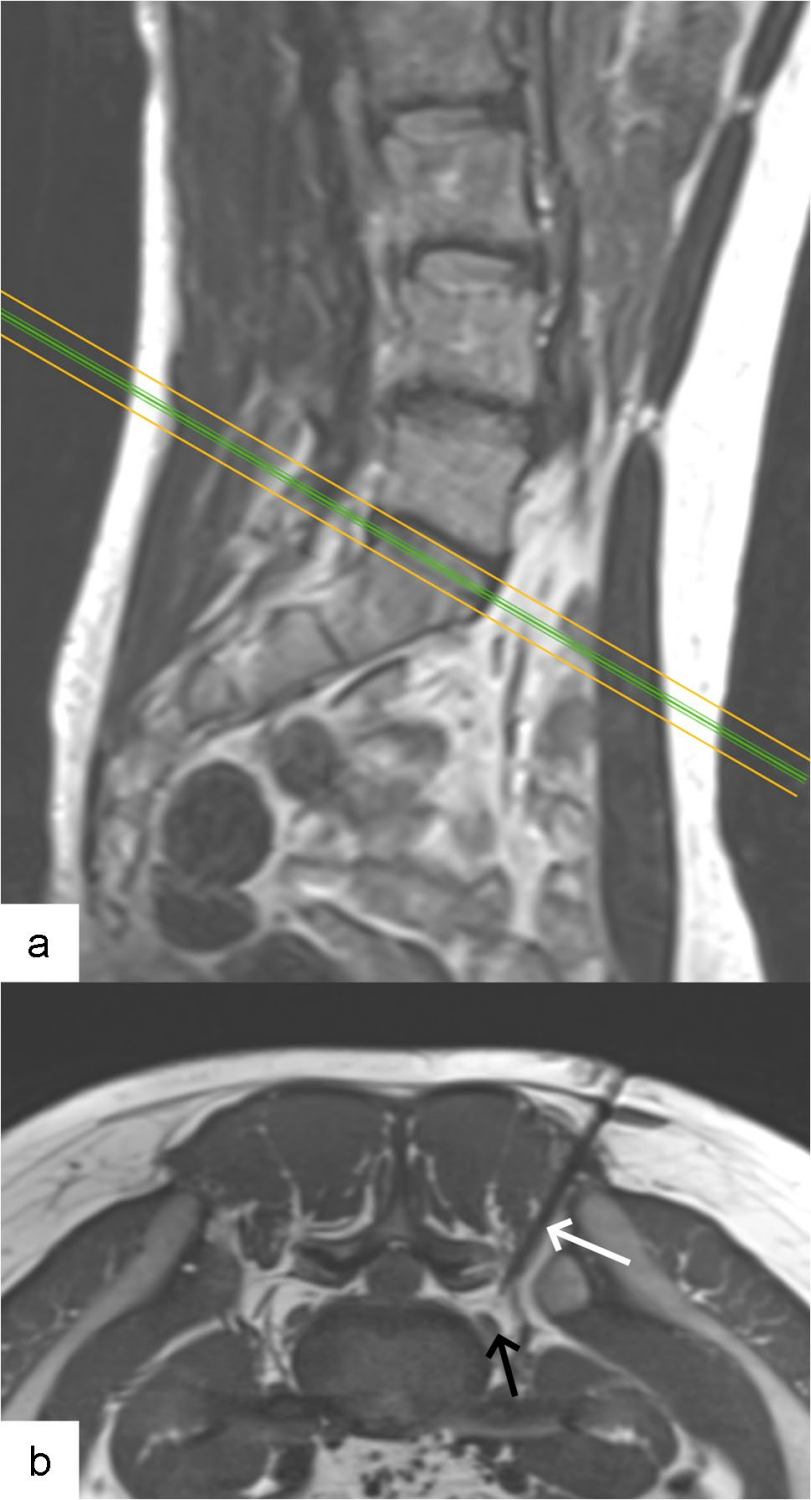
Non-contrast MRI scan obtained in nerve root infiltration in 24-year-old man. **a** Sagittal T2 turbo spin echo-weighted image showing oblique cranioventral access plane (green line) chosen to access L5 nerve within L5-S1 neural foramen. **b** Axial oblique proton density-weighted image showing 20G needle (white arrow) arriving behind left L5 nerve (black arrow)

**Fig. 4:**
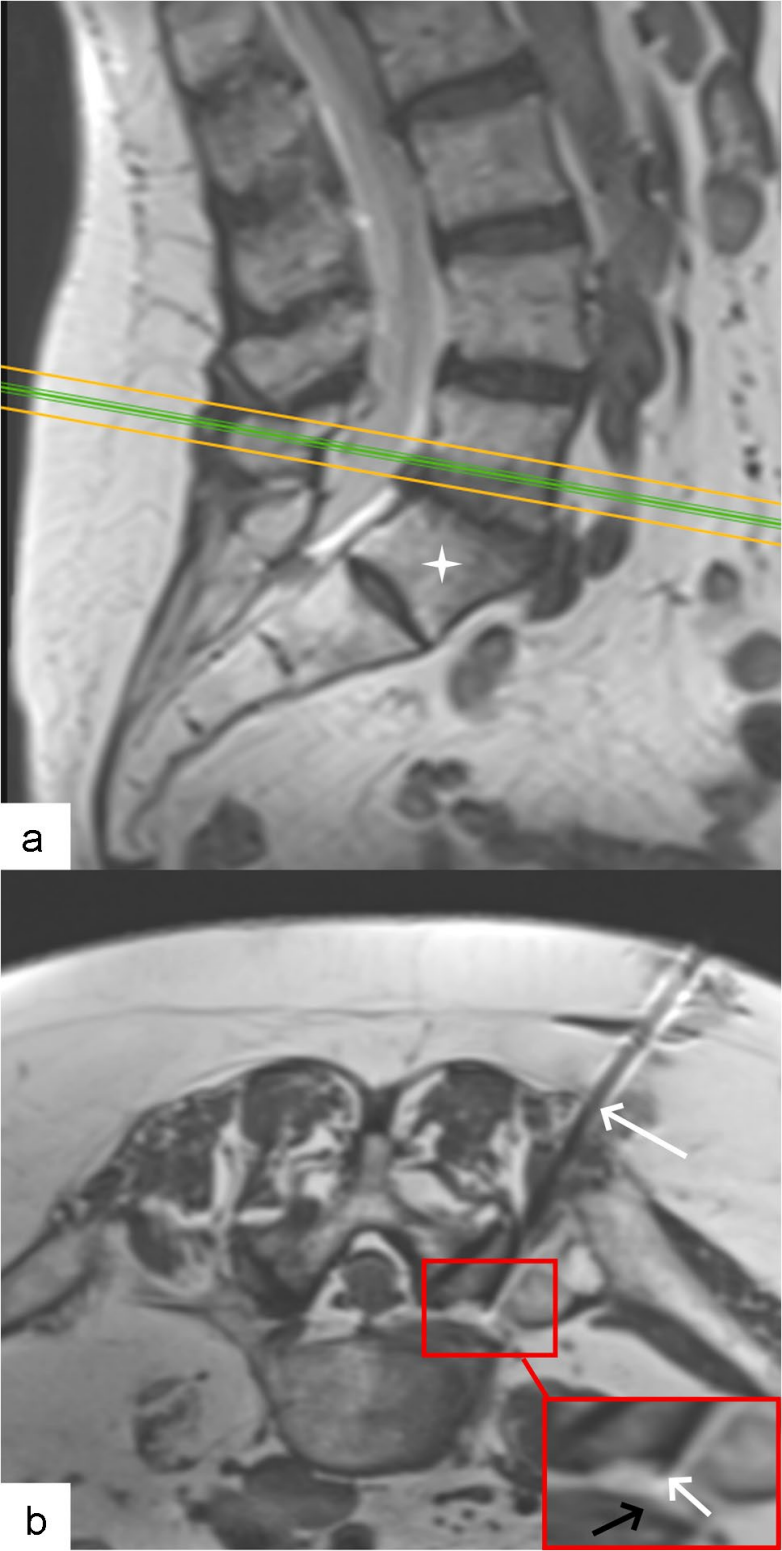
Non-contrast MRI scan obtained in nerve root infiltration in 70-year-old woman. **a** Sagittal T2 turbo spin echo-weighted image showing oblique cranioventral access plane (green line) chosen to access L5 nerve within L5-S1 neural foramen. Note transitional lumbo-sacral vertebra. **b** Axial oblique proton density-weighted image showing 20G needle (white arrow) with tip (arrowhead, inset) arriving behind left L5 nerve (black arrow, inset)

In case of lumbo-sacral transitional anomaly, the L5-S1 nerve root can be difficult to reach by a direct and strictly axial access. In this situation, MRI-guidance allowed for a three-dimensional planning and execution of access path (see Figs. 3 and 4 for examples from our own experience).

**Fig. 5:**
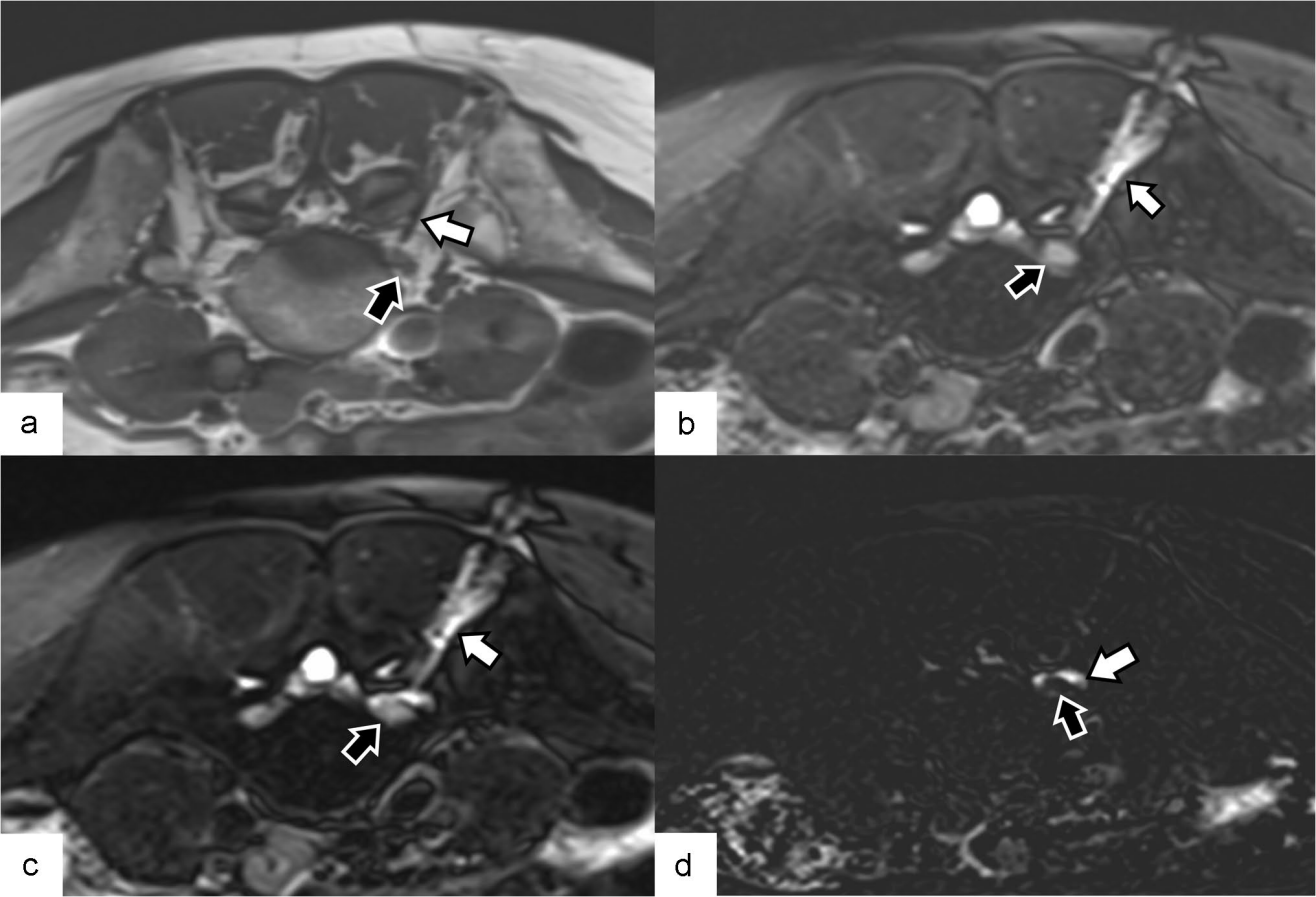
Non-contrast MRI scan performed during a nerve root infiltration procedure in a 41-year-old woman. **a** Axial proton density-weighted image illustrating a 22G needle (white arrow) positioned behind the left L5 nerve (black arrow). **b** Axial T2 turbo spin density (TSE)-weighted fat-saturated image revealing a fluid deposit (white arrow) surrounding the needle, resulting from the local anesthetic injection along the needle's path. The nerve appears hyperintense (black arrow). **c** At the same level, an axial T2 TSE-weighted fat-saturated image was acquired after injecting a small amount of sterile saline solution, which highlighted a minor fluid accumulation (white arrow) around the nerve (black arrow), thereby confirming the extravascular position of the needle tip. **d** By subtracting images **b** and **c**, the fluid deposit (white arrow) around the nerve (black arrow) became more obvious and helpful for visualization

Once the needle was in its final position, as close as possible to the root without touching it, a breathhold fat-saturated T2-weighted sequence was acquired. It was then followed by the injection of a small quantity of sterile normal saline solution, and then acquired again to compute a subtraction image showing the injected fluid clearly. Figure 5 shows the distribution of the saline solution around the nerve root, allowing to confirm the correct position of the infiltration needle and to estimate the direction of fluid diffusion. In addition, if the injected fluid is visible on the images, an extravascular position of the needle tip can be assumed. Table 1 shows the main acquisition parameters of the MRI sequences. In case of an excessive distortion of the fat-saturated T2 images by a total hip prosthesis, a free breathing STIR sequence was acquired instead. Finally, medication injections were performed around the nerve root.

**Table 1:** Acquisition parameters. *TR* repetition time, *TE* time to echo, *TA* acquisition time, *FOV* field of view, *TSE* turbo spin echo, *PD* proton density, *FS* fat saturated, *iPAT* integrated parallel acquisition technique

	TR (ms)	TE (ms)	Slice thickness (mm)	Slice interval (mm)	TA (s)	Matrix	FOV (mm)	Resolution	iPAT factor
T2 TSE	2300	103	2.5	2.75	9.4	512 × 512	300 × 300	512 × 512	2
PD	1030	9.1	2	2.4	13.6	336 × 256	315 × 240	336 × 256	2
T2 FS	2410	104	3	3.3	12.3	384 × 384	259 × 259	384 × 384	2

We generally used 1% lidocaine to anesthetize the needle path, followed by injections of 0.4% non-crystalline dexamethasone solution and 0.5% ropivacaine or bupivacaine.

### Clinical Data

Two aspects of the patient’s subjective pain were assessed. On the one hand, the locoregional pain as described in relation to the minimally invasive procedure itself was recorded for every patient, without counting the first injection for cutaneous anesthesia. On the other hand, chronic back pain evolution was the primary endpoint of this study. It was assessed a few days before the intervention, approximately 30 min after the infiltration on-site for every intervention, and then monthly for up to 5 months of follow-up.

A numeric rating scale (NRS), with 0 = no pain, from 1 to 3 = minor pain, from 4 to 6 = moderate pain, and from 7 to 10 = severe pain, was derived from the visual analog scale (VAS).

Patients received instructions from a staff member to quantitatively evaluate their pain level on the VAS at the aforementioned sampling points. During follow-up, patients were contacted by phone calls by the same staff member monthly from the first to the fifth month after the procedure, in order to record their pain level and other procedure-related information, such as the necessity for ultimate spinal surgery.

The success of the procedure was assessed by calculating the difference between the pain level experienced by the patient during the days before the procedure and the pain level experienced at each assessment by the physician during the 5 months following the procedure.

## Results

### Needle artifacts on magnetic resonance images

**Fig. 6:**
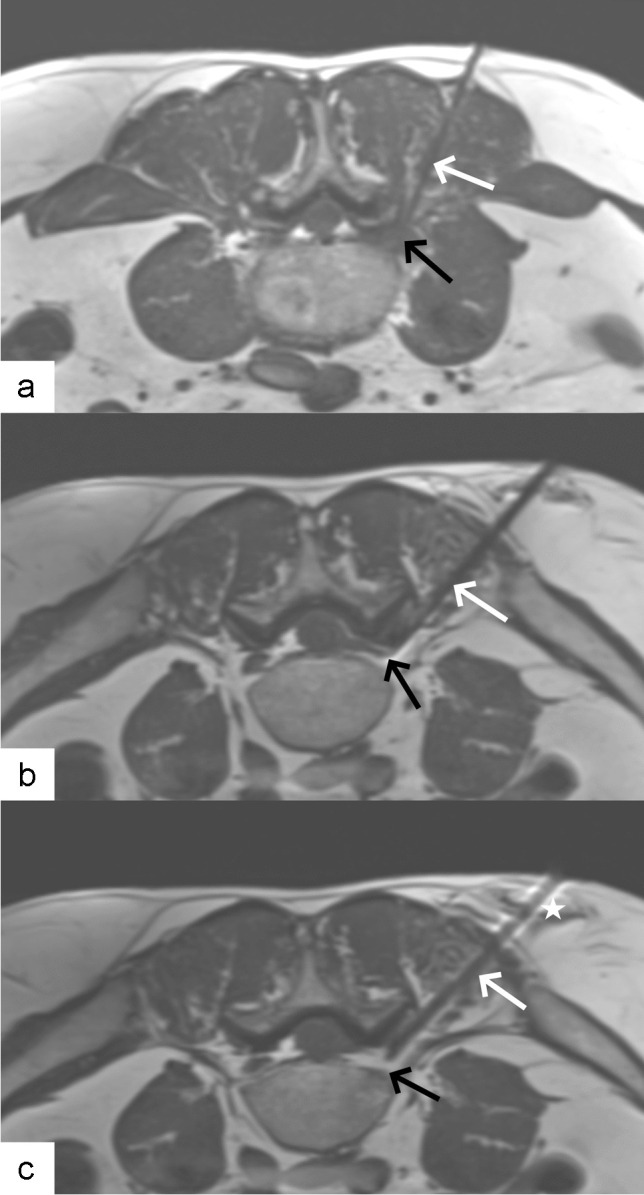
Non-contrast MRI scans obtained in nerve root infiltrations in 67-year-old man. **a** Axial proton density-weighted image showing 22G needle (white arrow) arriving behind fibrotic tissue (black arrow) surrounding left L4 nerve. **b**, **c** Axial proton density-weighted images showing 20G needle (white arrows) arriving behind left L5 nerve (black arrows). Hypointense needle artifact was reduced with usage of 22G needle (**a**) as compared with 20G needle (**b** and **c**). Note also hyperintense artifact presents on both sides of the needle in the phase-encoding direction (star in **c**)

Artifact measurements were performed on three patients who received infiltrations with a 22G needle and three patients who received infiltrations with a 20G needle. The average artifact width for the 20G needle was found to be 2.1 mm, whereas the average width for the 22G needle was 1.4 mm according to the method used in Scheffler et al*.* [22]. These findings demonstrated that the use of the 22G needle resulted in less pronounced artifacts, as depicted in Fig. 6. Based on our case series, we decided to adopt the 22G needle as an alternative to the 20G needle from that date onwards, when its higher relative flexibility allowed it, as it may cause challenges in steering during the procedure.

### Pain reduction

Complete follow-up responses were obtained for 39 procedures in 36 patients, with a median age of 61 years (range: 25–91). The locoregional pain related to the procedure itself was assessed as very low to mild (level 0 in five cases, level 2 in 15 cases, level 3 in 10 cases, level 4 in six cases).

**Fig. 7:**
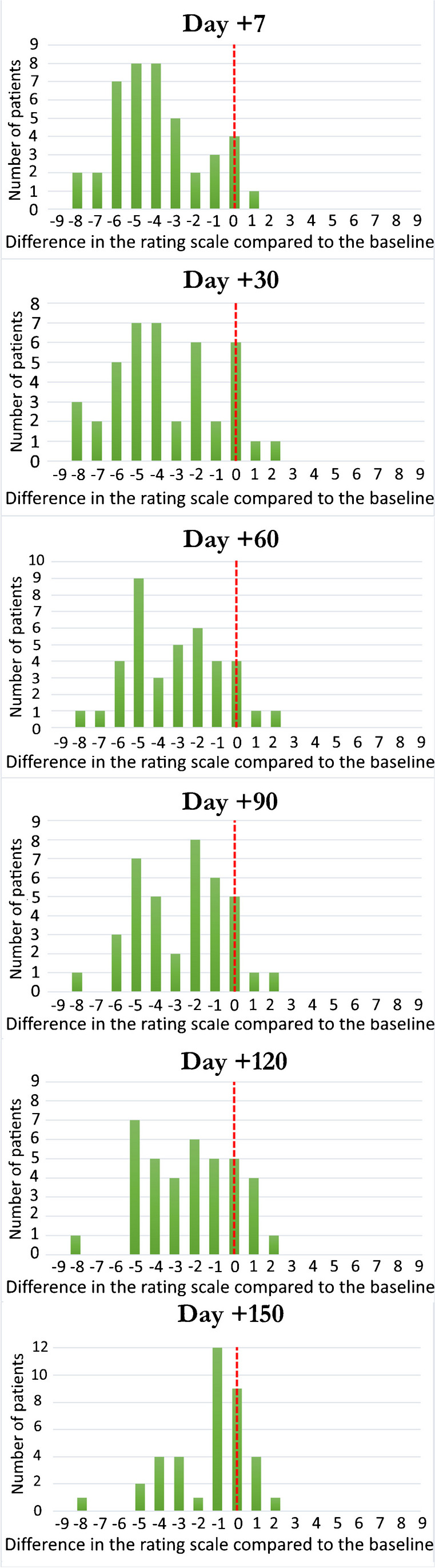
Difference in the numeric rating score at each assessment compared with the day before the procedure as a function of the number of treated patients

The pain level changes assessed by the NRS at the various time points are displayed in Fig. 7. Additionally, the average of this difference was calculated for the 39 procedures, along with the standard deviation (Table 2).

**Table 2:** Average and standard deviation of the difference between the level of pain experienced by the patient during the days before the procedure and the pain experienced at each assessment during follow-up. Numbers in the first line denote days in relation to the day of intervention. NRS, numeric rating scale; SD, standard deviation

	D + 3	D + 7	D + 30	D + 60	D + 90	D + 120	D + 150
Average NRS change	− 4.1	− 3.9	− 3.5	− 3.2	− 2.7	− 2.3	− 1.4
SD	2.3	2.3	2.6	2.4	2.3	2.3	2.1

Out of the 39 procedures, 36 procedures (92%) showed a pain reduction 1 week after the infiltration, 33 procedures (84%) 1 month later, 31 procedures (79%) 2 months later, 30 procedures (77%) 3 months later, 24 procedures (61%) 4 months later, and 20 procedures (51%) 5 months later (Table 3). Among the 40 patients in the study, four patients (10%) underwent surgery, denying any improvement felt 1 month after the procedure.

**Table 3:** Number of patients with a pain score reduction after the infiltration out of the 39 procedures. Numbers in the first line denote days in relation to the day of intervention

	D + 7	D + 30	D + 60	D + 90	D + 120	D + 150
Stable reduction of the pain score (patients)	36	33	31	30	24	20
(%)	92%	84%	79%	77%	61%	51%

One patient underwent four infiltrations (December 2017, August 2018, October 2019, and April 2021) and another patient underwent two infiltrations (November 2018 and July 2019). Each infiltration yielded similar results in pain reduction for both patients.

## Discussion

Chronic back pain is a significant global medical condition, which represents one of the leading reasons for seeking medical help and abstaining from work. It presents a major medical challenge for the foreseeable future. To the best of our knowledge, this study was the first to evaluate the feasibility, safety, and efficacy of 3 T MRI-guided lumbar or sacral nerve root infiltration with a 5-month follow-up.

In this analysis, we were also able to show the efficacy of this procedure under MRI guidance. A large fraction of patients (77%) reported a pain reduction 3 months after the intervention, with an average NRS change of 2.7 points. Moreover, 51% of patients reported a pain reduction after 5 months, with an average NRS change of 1.4 points. In our study, we did not perform a pain relief comparison versus CT or fluoroscopy guidance, but screening of literature data showed that our results are not inferior to that of CT or fluoroscopy procedures [28, 29].

In this study, MRI-guided infiltrations appeared to be as effective as CT-guided infiltrations, which may be due to the precise needle tip placement and the accurate distribution of saline solution, which corresponds to the drug diffusion area.

The safety profile of MRI-guided nerve root infiltration was excellent, with no reported complications during the procedures. This further supports the reliability of this technique in clinical practice.

Concerning workflow, we have had good experiences with utilizing a 22G needle instead of the 20G needle used in our previous study [22]. The systematic use of reconstructed subtraction images from T2 FatSat sequences increased the operator’s confidence. Six interventions were performed using a cranioventral obliquity approach for the needle path, and one case using caudoventral obliquity.

Procedure time optimization was observed, with a median time of 38 min in this study versus 51 min in our previous study [22]. These procedure times must be compared with the shorter durations of 20 to 30 min associated with CT and fluoroscopy in our institution. However, the learning curve demonstrated a procedure time reduction, with the last intervention requiring only 28 min compared with 70 min for the first one [22].

A small proportion of patients (10%) reported no pain improvement 1 month after the procedure, including two patients who received infiltrations on both sides of the vertebra. The obtained results offer significant insights for physicians. In cases where the steroid fails to produce the desired effect, which can occasionally occur, the injection of a local anesthetic around the nerve root can still offer crucial diagnostic information, despite its temporarily limited effectiveness. The images of these four patients were thoroughly examined to ascertain whether the needle’s placement or the product diffusion could have caused the failure. Analysis of the needle position data revealed that, in each patient, the needle was located approximately 1.8 mm to 2.4 mm away from nerve the root, aligning with findings from the other cases investigated by Scheffler et al. [22].

Although our technical and clinical results are encouraging, this study has some relevant limitations. First, the patient number was relatively small, which may have prevented some findings from being identified. Second, this study showed that even when the procedure is performed within a standardized setting, patients do not always respond equally to nerve root infiltrations. Some patients did not feel any improvement after the infiltration, whereas for others, pain reduction was still effective 5 months after the procedure. This can be explained by two factors: the first one being the underlying pathology (neuro-foraminal narrowing, hernia, possible non-mechanical inflammation), which seems to play a crucial role regarding therapeutic efficacy; the second factor concerns secondary patient care such as physiotherapy, which has positive effects on pain reduction. Therefore, further limitations of this study are that it does not consider the different pathologies of the patients and the secondary care received.

In the future, the development of metal artifact reduction (MAR) sequences holds potential for further enhancing the accuracy and efficacy of MRI-guided nerve root infiltrations. By mitigating the impact of metallic artifacts resulting from implanted devices, MAR sequences enable improved visualization and precise needle placement [30]. However, it is important to note that these sequences require substantial time for completion. This improvement may contribute to better patient outcomes and expand the applicability of MRI-guided procedures in the field of neuroradiology.

Another area for future development could be to compare the immediate to mid-term effects (resulting from local anesthesia, injected last) with the mid- to long-term effects (resulting from steroids, injected first). Infiltrations are often requested for patients who are highly likely to eventually undergo surgery. Exploring the possibility that a positive immediate effect may serve as a predictor for a favorable mid- to long-term outcome would be intriguing and valuable. Further research is warranted to explore these possibilities and optimize chronic back pain management.

## Conclusion

In this study, we evaluated the use of 3 T MRI-guided nerve root infiltration for radicular pain management, particularly in the lumbar spine. Our findings demonstrated that MRI-guided nerve root infiltration is a safe, feasible, and precise technique in well-selected patients, with a high rate of pain improvement. The absence of complications and the overall positive response rate of 61% after 4 months and 51% after months indicate that this technique holds promise as an effective treatment option for radicular pain. Our study highlighted that the use of MRI guidance allows for accurate needle placement and precise targeting of the affected nerve root, resulting in significant pain reduction for most patients. The addition of MRI-guided nerve root infiltration to the existing range of treatment options offers a valuable tool for chronic back pain reduction. Indeed, this technique can expand the therapeutic armamentarium available to clinicians and enhance patient care by providing a safe and effective alternative.

In conclusion, our study highlighted the potential of MRI-guided nerve root infiltration as a valuable technique in the management of radicular pain. With its favorable safety profile, high rate of pain improvement, and precise guidance, this technique can complement existing approaches and contribute to the comprehensive treatment of chronic back pain. Further research and larger-scale studies are warranted to validate our findings and explore the long-term outcomes and potential extended applications of MRI-guided nerve root infiltration in the field of neuroradiology.

## Data Availability

The data that support the findings of this study are not openly available but are available from the corresponding author upon reasonable request. Data are located in the picture archiving and communication system (PACS) at Geneva University Hospitals.

## Abbreviations

MRI: Magnetic resonance imaging
CT: Computed tomography
PD: Proton density
MAR: Metal artifact reduction
TSE: Turbo spin echo
STIR: Short time inversion recovery
